# Primary age-related tauopathy and the amyloid cascade hypothesis: the exception that proves the rule?

**DOI:** 10.29245/2572.942x/2016/6.1059

**Published:** 2016

**Authors:** John F. Crary

**Affiliations:** Department of Pathology, Fishberg Department of Neuroscience, Friedman Brain Institute, Ronald M. Loeb Center for Alzheimer’s Disease, Icahn School of Medicine at Mount Sinai, USA

## Abstract

Extensive data supports the amyloid cascade hypothesis, which states that Alzheimer’s disease (AD) stems from neurotoxic forms of the amyloid-beta (Aβ) peptide. But the poor correlation between Aβ plaques and neurodegeneration/cognitive impairment, the spaciotemporal disparity between Aβ and tau pathology, and the disappointing results following several large clinical trials using Aβ-targeting agents are inconsistent with this explanation. The most perplexing inconsistency is the existence of AD-type dementia patients that develop abundant neurofibrillary tangles that are indistinguishable from those in early to moderate-stage AD in the absence of compelling evidence of amyloid toxicity. This neuropathological phenotype, which is distinct from other diseases with tangles, represents a conceptual disconnect, because it does not fall within any previously established category of tauopathy and ostensibly invalidates the amyloid cascade hypothesis. Instead, recent efforts have led to consensus criteria for a new alternative diagnostic category, which presupposes that these tangle-only dementia patients represent extreme examples of a distinct primary age-related tauopathy (PART) that is universally observed, albeit to varying degrees, in the aging brain. The cause of PART is unknown, but sufficient evidence exists to hypothesize that it stems from an Aβ-independent mechanism, such as mechanical injury. Should the PART hypothesis withstand further experimental testing, it would represent a shift in the way a subset of subjects with AD neuropathological change are classified and has the potential to focus and reaffirm the amyloid cascade hypothesis.

## Emergence of the amyloid cascade hypothesis

In 1906, Alois Alzheimer observed a progressive dementing illness in a 55-year-old woman^1^. Using a new Bielschowsky silver stain, he observed the co-occurrence of amyloid plaques, previously described by Blocq and Marinescu, alongside a distinctive new lesion, termed the neurofibrillary tangle (NFT). The moniker Alzheimer’s disease (AD), promulgated by Emil Kraepelin, was originally applied strictly to early-onset (i.e., pre-senile) dementia patients. But later, the term was broadened to encompass all dementia patients where plaques and tangles could be observed.

A series of findings provided the foundation for the amyloid cascade hypothesis, which maintains that increased Aβ is the root cause of both rare familial and the more common sporadic forms of AD^2^. All other features (e.g., synaptic dysfunction, neurodegeneration, and cognitive impairment) were considered secondary, including NFT. Mutations in genes that influence Aβ production (i.e., presenilin 1, presenilin 2 and the amyloid precursor protein) cause familial early-onset AD^3-6^. Patients with trisomy 21 (Down syndrome) have triplication of the *APP* gene and essentially all develop AD^7^. Further, *APOE* ε4, the strongest risk allele for late-onset AD, is strongly associated with Aβ deposition^8^. Together with the argument that all patients with AD have Aβ deposition, though tautological because the diagnostic criteria require plaques, reinforced the assertion that Aβ is the primary cause. The hypothesis has undergone revisions, but in essence remains intact^9,10^.

## Challenges in applying the amyloid cascade hypothesis

Applying the framework provided by the amyloid cascade hypothesis to diagnosing and treating AD has proven problematic^11-16^. Early neuropathological criteria for diagnosing AD focused on Aβ burden^11^, but this strategy was not optimal given that total Aβ plaques correlate poorly with cognitive impairment and neuronal loss^17^. Many investigators feel that the relevant lesion is the neuritic amyloid plaque, a distinct subtype that is differentiated by the presence of tau-positive neurites. The CERAD criteria, based on semiquantitative assessments of neuritic amyloid plaques, reflects this position^18^. To address the poor correlation between plaques and cognitive impairment, the CERAD criteria also consider AD as a clinicopathological diagnosis, requiring premortem evidence of cognitive dysfunction for a diagnosis of definite AD. Some investigators maintain that AD is a disease of both plaques and tangles. Thus, the NIA-Reagan neuropathological criteria were developed^19^. These 1997 criteria deploy both CERAD and the Braak NFT staging system, an approach based on the supposition that tau pathology progresses in a stereotyped hierarchical manner from the entorhinal cortex, through medial temporal lobe structures and eventually diffusely throughout the neocortex^20,21^. This system was later revised to incorporate early brainstem pathology. Another staging scheme for amyloid plaque progression, the Thal phase, assesses the progression of amyloid from neocortex, to limbic structures and ultimately cerebellum and brainstem. The recent NIAAA “ABC” system deploys Thal, Braak NFT and CERAD assessments. These 2012 criteria notably abandon the requirement of a premortem clinical dementia diagnosis, a requirement that was suggested to impede efforts to study patients with early presymptomatic and prodromal disease.

Several large phase III clinical trials of therapeutics targeting Aβ have failed due to lack of efficacy, prompting reflection as to whether the amyloid cascade hypothesis is invalid^22,23^. The reason for these failures remain unclear, but some investigators have cited these failed trials as evidence refuting the amyloid cascade hypothesis. Other investigators and pharmaceutical companies have concluded that the design of the trials, which failed to confirm target engagement, were the reason. Another possibility is that Aβ triggers a complex neurodegenerative cascade with a late amyloid-independent phase^24^. The future success of an Aβ-targeting agent is required for final validation of the amyloid cascade hypothesis.

## Advances in neuropathological sub-classification of dementia singles out tangle-only pathology

While the heterogeneity of dementing illnesses has complicated efforts to understand the relationship between Aβ and cognitive failure, recent progress in understanding non-AD dementias has put AD into sharper focus. Some of pathologies are more readily differentiated from AD neuropathologically, such as vascular dementia, but this can be difficult to quantify. The TDP-43 proteinopathies (e.g., frontotemporal lobar degeneration, amyotrophic lateral sclerosis and hippocampal sclerosis-aging/cerebral age-related TDP-43 with sclerosis (CARTS) are largely devoid of Aβ and tau pathology^25^. The more closely overlapping “plaque-only dementia” cases were found to largely represent an α-synucleinopathy (i.e., diffuse Lewy body disease)^26^.

The discovery of *MAPT* mutation in rare families demonstrating that tau dysfunction alone is sufficient to cause neurodegeneration represented a major breakthrough. But such cases are rare with a distinct frontotemporal dementia syndrome^27^. The degenerative movement disorders (e.g., Parkinson’s disease, progressive supranuclear palsy and corticobasal degeneration) are readily differentiated from AD clinically and neuropathologically by differences in regional vulnerability and distinctive glial pathology^28^. Chronic traumatic encephalopathy (CTE), first described in professional boxers, has received greater scrutiny because of the recent link to contact sports, particularly American football, and recent consensus criteria have greatly improved our ability to recognize this pathology^29^.

Another pattern of degeneration, however, which has been variably called tangle-only dementia (TOD), neurofibrillary tangle predominant senile dementia, tangle-dominant dementia, among many other monikers, has received far less attention^30^. But large dementia autopsy series designed to advance our understanding of AD have allowed TOD to come into sharper focus and culminated in the development of a new diagnostic category termed primary age-related tauopathy (PART). New consensus criteria place TOD on a continuum with age-related tangles, that are universally observed in aged brains^31^. Considerable evidence (see below) indicates that subjects with PART have a distinct constellation of features that sets them apart from classical “plaque and tangle” AD and other tauopathies. Studying these differences may provide clues to the pathogenesis of tauopathies and refine the amyloid cascade hypothesis.

## Neuropathological and clinical features of PART

The NFT in PART are essentially identical to those observed in AD^31^. They are composed of similar tau isoforms (3 and 4 repeat), form paired-helical filaments, and are concentrated within neurons. The NFT in PART are localized to the medial temporal lobe in a distribution corresponding to up to Braak IV. NFT in this distribution can be observed in subjects with normal cognition, mild cognitive impairment and dementia. In cognitively normal elderly subjects, autopsy studies have demonstrated that medial temporal lobe NFT are essentially universal and in a more limited distribution in many younger individuals. In demented subjects, approximately 2-10% of subjects display such tangles without significant amyloid deposition^31^. The proportion of subjects with age-associated memory impairment or mild-cognitive impairment in association with PART might be high. There are a very large number of subjects within the biomarker/imaging-defined category of suspected non-amyloid pathophysiology (SNAP) which has overlap with PART^32^. Finally, given that Aβ-deposition is commonly encountered in cognitively normal subjects, “benign Aβ” deposits might be masking an underlying tauopathy in some patients leading to reduced prevalence estimates. Methods for differentiating PART tangles and AD tangles (e.g., biochemical or immunohistochemical markers) would be extremely helpful for answering this question.

Tangle-only dementia (TOD) was first described in a series of patients with clinical features that were very similar to those of classical AD^30^. While this category likely included some subjects with other dementing tauopathies, a large proportion have PART as a primary pathological dementing process. Key features of TOD that differentiate such subjects are older age of death, a female predominance and a somewhat milder amnestic dementia. While the degree of cognitive impairment can be severe in some subjects, it is our impression that many of such subjects have mixed pathology, often vascular disease (unpublished observations). Other psychotic symptoms have been observed, but this needs further clarification. More work needs to be done to further delineate the clinical and neuropathological spectrum of PART.

## Beyond Aβ: drivers of PART

What exactly PART represents has been the matter of debate, with various investigators considering it an AD variant, a frontotemporal dementia variant, or normal (or “pathological”) aging. Toxins and infectious causes are also possible, but less likely^33,34^. Currently, the evidence fails to support a role for Aβ toxicity in PART. Subjects with PART have no Aβ deposition, no association with *APOE* ε4, and biochemistry fails to show evidence of increased soluble Aβ in PART brain parenchyma^35^. Another hypothesis, that postulates early Aβ-dependent and subsequent Aβ-independent phases, is theoretically possible^24^. However, evidence supporting this notion is lacking and how it would be tested experimentally is unclear. Thus, Aβ-dependent mechanisms are unlikely to play a pathogenic role in PART.

Neither imbalances in tau splicing nor mutations in the tau gene have been found in PART^35^. However, the microtubule-associated protein tau gene (*MAPT*) H1 haplotype, a risk allele that is associated with other tauopathies without a coding region mutation, has been investigated, suggesting an association with the H1 haplotype^35,36^. Together, an emerging theme suggests that PART dementia patients have genetic features that protect from amyloid accumulation but also alleles that serve as risk factors for tauopathy. Future studies are required to fully address this possibility.

The possibility that PART is a form of pathological brain aging deserves attention. Mechanical injury in the form of mild yet repetitive traumatic brain injury (TBI) is an established trigger for tauopathy in chronic traumatic encephalopathy (CTE) in elite athletes and boxers^37^. While subjects develop PART in the absence of documented TBI, the hypothesis that these tangles are caused by very mild repetitive “wear and tear” type injury can be supported by three arguments. First, the geometry of the human central nervous system is such that foci of mechanical stress concentration are predicted to include the medial temporal lobe and basal forebrain. Second, the presence of an uncal notch in the medial temporal lobe that overlies the transentorhinal cortex is very common even in the absence of cerebral edema^38^, providing direct physical evidence that this site is a focus of stress concentration. Third, patients with known repetitive mechanical brain injury (i.e., CTE) develop tangles in an overlapping distribution, however more widespread and of greater magnitude^39^. Thus, it is reasonable to hypothesize that the cause of PART is a very mild repetitive mechanical “wear and tear” type of age-related injury.

## Criticism of the PART hypothesis

Some investigators have suggested that PART and AD should not be differentiated^40,41^. Instead, they maintain that an analytical approach should be used. This argument, based on the frequency of amyloid and tau lesions in the brain over the aging spectrum, presupposes that all PART subjects will eventually develop amyloid plaques had they lived long enough. The fact that the burden of amyloid pathology decreases in centenarians does not support this conclusion^42^. Also, PART dementia subjects are generally very old, have end stage tauopathy with frequent ghost tangles, and marked atrophy and gliosis, but in a restricted in distribution, suggesting that they are at the end of their disease course, rather than the beginning and argues against the hierarchical progression model in these patients. Another criticism of the PART hypothesis is that the cause of both PART and classical late-onset AD continue to be unknown. Without causality established, it is impossible to know with absolute certainty that these represent two distinct processes rather than different manifestations of a common “dual” pathway as has been hypothesized^43^.

## Conclusion

Sufficient evidence exists to argue that PART represents a distinct pathological category, and the rapid adoption of the terminology indicates that practicing neuropathologists find utility in the terminology. This new conceptual framework will provide physician scientists and basic researchers with a new approach to stratifying subjects with AD neuropathological change, especially at the earliest stages when there is the highest likelihood of interventions achieving therapeutic success. Should PART be an Aβ-independent cause of AD-type dementia, it may be an exception that helps to establish the validity of the amyloid cascade hypothesis.
